# Ferroptosis and its Role in Gastric Cancer

**DOI:** 10.3389/fcell.2022.860344

**Published:** 2022-06-30

**Authors:** Renjun Gu, Yawen Xia, Pengfei Li, Defang Zou, Keqin Lu, Lang Ren, Hongru Zhang, Zhiguang Sun

**Affiliations:** ^1^ Nanjing University of Chinese Medicine, Nanjing, China; ^2^ Jiangsu Provincial Second Chinese Medicine Hospital, The Second Affiliated Hospital of Nanjing University of Chinese Medicine, Nanjing, China; ^3^ Jiangsu Key Laboratory for Pharmacology and Safety Evaluation of Chinese Materia Medica, School of Pharmacy, Nanjing University of Chinese Medicine, Nanjing, China; ^4^ Jiangsu Collaborative Innovation Center of Traditional Chinese Medicine Prevention and Treatment of Tumor, Nanjing University of Chinese Medicine, Nanjing, China; ^5^ Department of Clinical Laboratory, Jiangsu Province Hospital of Chinese Medicine, Affiliated Hospital of Nanjing University of Chinese Medicine, Nanjing, China; ^6^ School of Basic Medical Sciences, Nanjing University of Chinese Medicine, Nanjing, China

**Keywords:** ferroptosis, iron, gastric cancer, ROS, microenvironment, drug resistance

## Abstract

Gastric cancer (GC) is the fifth most common cancer and the third leading cause of cancer-related deaths worldwide. Currently, surgery is the treatment of choice for GC. However, the associated expenses and post-surgical pain impose a huge burden on these patients. Furthermore, disease recurrence is also very common in GC patients, thus necessitating the discovery and development of other potential treatment options. A growing body of knowledge about ferroptosis in different cancer types provides a new perspective in cancer therapeutics. Ferroptosis is an iron-dependent form of cell death. It is characterized by intracellular lipid peroxide accumulation and redox imbalance. In this review, we summarized the current findings of ferroptosis regulation in GC. We also tackled on the action of different potential drugs and genes in inducing ferroptosis for treating GC and solving drug resistance. Furthermore, we also explored the relationship between ferroptosis and the tumor microenvironment in GC. Finally, we discussed areas for future studies on the role of ferroptosis in GC to accelerate the clinical utility of ferroptosis induction as a treatment strategy for GC.

## Introduction

Gastric cancer (GC) is the fifth most common cancer and the third leading cause of cancer-related deaths worldwide (Smyth et al., 2020). Some of the risk factors for this disease include *Helicobacter pylori* infection, age, high salt intake, and unhealthy diet (Chen et al., 2021a). GC is commonly treated with surgery (Mihmanli et al., 2016). However, the associated expenses and post-surgical pain impose a huge burden on GC patients. Several studies which focused on identifying molecular signatures and genetic alterations in GC in order to improve treatment selection and aid drug development have already been conducted (Lordick et al., 2017). However, the underlying mechanisms in disease progression are still unclear. Thus, an in-depth understanding of the GC pathobiology will not only facilitate the identification of new drug targets but also provide help in the development of new clinical treatment strategies.

Ferroptosis is a relatively new form of programmed cell death, which was first described in 2012 (Dixon et al., 2012). Several studies have implicated the contribution of ferroptosis in the progression of multiple diseases (Galluzzi et al., 2018; Stockwell et al., 2020), including GC (Jiang et al., 2021a; Liu G. et al., 2021). In this review, we summarized the relationship between ferroptosis and gastric cancer. Furthermore, we suggested that effective regulation of iron metabolism may provide a novel strategy for treating gastric cancer.

## Iron Metabolism in Gastric Cancer

Iron is an indispensable molecule in almost all living organisms. Iron-containing enzymes are involved in many physiological activities (Dixon and Stockwell, 2014), such as cellular metabolism, oxygen transport, DNA synthesis, energy production, and cellular respiration. Aside from its roles in various life processes, the catalytic form of iron can also catalyze the formation of reactive oxygen species (ROS) in oxygen-rich environments. Interestingly, iron and ROS can initiate and mediate cell death in several organisms and disease states (Fischbacher et al., 2017). In addition, ROS can also affect several processes, such as cell survival, proliferation, and differentiation through multiple signaling pathways (Lambeth and Neish, 2014). Although low cellular ROS levels are beneficial to some extent, it has also been found to result in base modification and DNA strand breaks (Inoue and Kawanishi, 1987; Dizdaroglu et al., 1991). These findings hint at the potential contribution of free radical-induced DNA damage in the etiology of numerous diseases, including cancer (Dizdaroglu and Jaruga, 2012). Consistent with this idea, extensive studies have shown that poor regulation of iron metabolism is associated with many diseases, including atherosclerosis, neurodegenerative disorders, and cancer (Lambeth and Neish, 2014; Zhou et al., 2018; Vinchi et al., 2019).

Iron levels and stomach health are closely interrelated. For example, several iron-related conditions, such as unexplained iron deficiency, idiopathic thrombocytopenic purpura, and anemia, were found to be associated with *H. pylori* infection (Hagymási and Tulassay, 2014; Durazzo et al., 2021). Iron homeostasis has also been implicated in cancer development. In one study, iron oxidation has been shown to contribute to tumor formation and subsequent cancer development (Torti and Torti, 2013). On the other hand, several studies reported that iron deficiency may enhance the risk of developing cancer (Janssen et al., 2020). Anemia, low serum ferritin levels, and autoimmune gastritis–related iron malabsorption were identified as risk factors associated with gastrointestinal tumors and GC (Nomura et al., 1992; Cover et al., 2013; Kamada et al., 2021). Consistent with these studies, *in vivo* data from rodent models show that iron deficiency may contribute to early progression of gastrointestinal tumors (Prá et al., 2009). Taken together, these studies highlight the significant role of iron in the development of various gastrointestinal malignancies, and the potential value of iron regulation as a treatment strategy (Palzer et al., 2021).

## Mechanism of Ferroptosis

Ferroptosis is a unique form of cell death (Kerr et al., 1972; Jacobson and Raff, 1995; Christofferson and Yuan, 2010; Shimada et al., 2016). Some protein modulators, such as p53, can exert their physiologic functions either through apoptosis or ferroptosis (Jiang et al., 2015). Similarly, several small molecules can initiate cell death *via* specific molecular events related to either ferroptosis, apoptosis, or necrosis (Kerr et al., 1972; Schweichel and Merker, 1973; Yang and Stockwell, 2008; Dixon et al., 2012; Shimada et al., 2016). The activation of distinct pathways suggests that the molecular mechanism involved in ferroptosis differs from those of apoptosis and necrosis (Dixon et al., 2012; Dong et al., 2015; Shimada et al., 2016; Torii et al., 2016).

Iron accumulation is the first step in ferroptosis (Galluzzi et al., 2015). Free ferric iron (Fe^3+^) in the blood conjugates with transferrin proteins. The iron-bound transferrin molecules are then captured by the transferrin receptors present on the cell membrane and enter the cell through endocytosis (Gao et al., 2019). Reducing proteins, such as six transmembrane proteins of prostate 3 (STEAP3), reduces Fe^3+^ to its highly reactive ferrous ion (Fe^2+^) form. Upon conversion, Fe^2+^ is transported from the endosomes to the cytoplasm and is included to the labile iron pool. To protect the cells and tissues from iron-mediated damage, excess Fe^2+^ in the iron pool is stored in ferritin, while the remaining Fe^2+^ can be pumped out of the cell through ferroportin molecules on the cell membrane (Yang et al., 2016; Hassannia et al., 2019). Under normal conditions, intracellular iron concentrations remain stable (Yamaguchi et al., 2021). However, in cases of iron overload, excessive Fe^2+^ is produced within the cell. The accumulation of intracellular Fe^2+^ further leads to the production of Fe^3+^ and ROS through the Fenton chemical reaction (Talvenmäki et al., 2019). In addition, excess Fe^3+^ can also be reduced to Fe^2+^ through the Haber–Weiss reaction (Kehrer, 2000). Furthermore, under stress conditions, ferritin can self-degrade into Fe^2+^ through iron autophagy (Talvenmäki et al., 2019). Collectively, these processes can lead to ferroptosis, which in turn induces the formation of more ROS. Excessive ROS can damage biofilms, proteins, and nucleic acids eventually leading to cell death (Kehrer, 2000).

**FIGURE 1:**
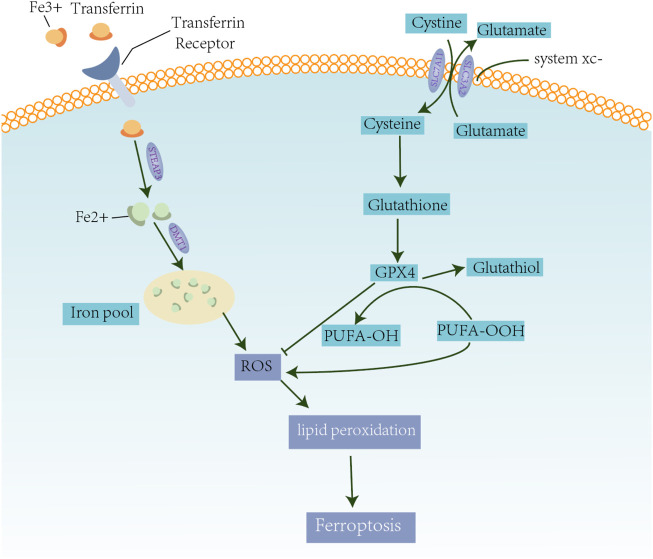
Mechanisms of ferroptosis. Excess iron is related to lipid peroxidation and abnormal iron metabolism of mercaptan, which induces the production of ROS. On the one hand, circulating iron in the form of Fe^3+^ binds to the transferrin receptor and enters the cell. Iron oxide reductase, STEAP3, reduces Fe^3+^ to Fe^2 +^, which is transported to the iron pool through DMT1 to induce the formation of ROS. Finally, it promotes lipid peroxidation and causes ferroptosis. On the other hand, the Xc system transports intracellular Glu to the extracellular space and extracellular cystine simultaneously into the cells, which is then transformed into cysteine for GSH synthesis. GPX4 converts -OOH to -OH in polyunsaturated fatty acid (PUFA) to reduce ROS accumulation.

The high intracellular ROS and free radical levels are usually controlled by cells through the actions of antioxidants such as glutathione (GSH) and glutathione peroxidase 4 (GPX4) (Aldini et al., 2018). However, in some cases, glutathione and GPX4 are used up by the cells in other processes, such as in regulating amino acid metabolism. The intracellular levels of glutathione are also affected by amino acid availability. Increased glutamine decomposition may affect the synthesis of glutathione and cause a cell death event similar to a GSH consumption–induced ferroptosis (Linkermann et al., 2014a; Yang et al., 2014). Furthermore, since GPX4 converts the potentially toxic lipid hydroperoxides (L-OOH) to non-toxic lipid alcohols (L-OH) (Ursini et al., 1982), the inactivation of GPX4 can ultimately cause cell death (Linkermann et al., 2014b) (Figure 1).

## Ferroptosis in Gastric Cancer

### Proliferation, Invasion, and Metastasis of Gastric Cancer

The proliferation, invasion, and metastasis of tumor cells are crucial events in the occurrence and development of malignant tumors. These cell activities lead to varying degrees of clinical responses (Machlowska et al., 2018). Like in other cancer types, GC develops from preneoplastic and early neoplastic precursor lesions (Song et al., 2015). These lesions may develop into tumors when the rate of cell proliferation is faster than cell death (Ginsberg et al., 1996; Li et al., 2018). Early stages of GC are characterized by good prognosis with 5-year survival rates reaching >90%; however, most patients are already in the advanced stages of the disease upon initial diagnosis (Tan, 2019). To date, curative surgical resection procedure is the only available treatment for GC (Santoro et al., 2014). Unfortunately, the metastasis of malignant tumors often causes treatment failure (Coburn et al., 2018; Hatta et al., 2020). Tumor invasion and metastasis refer to cellular events when malignant tumor cells continue to grow from the primary site into other sites through lymphatic, vascular, or the body cavity routes. The origin of tumor cells, genetic variations, circulatory mode, and the physiological structure of the metastatic organ determine the specific sites for distant metastasis (Jin et al., 2014).

Several studies try to identify other potential candidates for GC treatment (Table 1). One of these substances, Tanshinone IIA, can inhibit tumor proliferation and metastasis by increasing the level of lipid peroxides and decreasing that of glutathione in the GC cells (Ni et al., 2021a). Another extract, *Actinidia chinensis* (Planch) exerts anti-proliferation and anti-migration effects on GC cells. Additionally, it can significantly downregulate the expression of GPX4 in a dose-dependent manner (Gao et al., 2020). On the other hand, physcion 8-O-β-glucopyranoside displays antitumor effects in several cancer types, and it induces ferroptosis by regulating the miR-103a-3p/GLS2 axis in GC (Niu et al., 2019).

**TABLE 1:** Candidate substances and genes for inducing ferroptosis in gastric cancer.

Substances and Genes	Target/Function	Mechanism
*Actinidia chinensis* Planch (Gao et al., 2020)	GPx4, SLC7A11	Induces ROS accumulation
Tanshinone IIA (Guan et al., 2020)	Ptgs2, Chac1, p53, xCT	Tanshinone IIA upregulates p53 expression and downregulates xCT expression; Tan IIA decreases intracellular glutathione and cysteine levels and increases the levels of intracellular ROS.
Tanshinone IIA (Ni et al., 2021a)	SLC7A11	Induces ROS accumulation
Physcion 8-O-β-glucopyranoside (Niu et al., 2019)	GLS2	Induces ROS accumulation
Erastin (Sun et al., 2020)	Mitochondrial dysfunction	Induces ROS accumulation
Erastin (Chen L. et al., 2020; Mao et al., 2021)	SLC7A11	Induces ROS accumulation
Cysteine Dioxygenase 1	GPX4, maintains stability of mitochondrial morphology	Mediates erastin (Hao et al., 2017); induces ROS accumulation
Exosomes miR-522 (Zhang et al., 2020)	ALOX15	Leads to ALOX15 suppression, decreased lipid-ROS accumulation in cancer cells, and ultimately results in decreased chemosensitivity
SIRT6 (Cai et al., 2021)	GPX4	Inhibits GPX4 activity, induces ROS accumulation
CPEB1 (Wang J. et al., 2021)	Gpx4	Induces ROS accumulation
MiR-375 (Ni et al., 2021b)	SLC7A11	Induces ROS accumulation

Consistent with the studies on the relationship of iron and GC, ferroptosis has also been found to be closely related to the proliferation, invasion, and metastasis in GC (Chen et al., 2021a; Huang et al., 2021). However, the predictive role of ferroptosis in GC remains elusive (Shao et al., 2021). Thus, understanding the processes underlying ferroptosis is promising for the development of cancer treatment strategies.

## Tumor Microenvironment in Gastric Cancer

A tumor is closely connected with where it arises and develops in the organism (Chen et al., 2015). Tumor cells in tumor microenvironment (TME) play an active role in the disease progression (Bubnovskaya and Osinsky, 2020; Jiang et al., 2020). The TME favors the growth and expansion of cancer cells (Oya et al., 2020; Rojas et al., 2020). Interestingly, tumor cells and their surrounding microenvironment can be shaped by varying degrees of ferroptosis activation (Zavros, 2017; Xiao et al., 2021). It has been found that ferroptosis serves as an important factor in the formation of the TME in GC (Jiang et al., 2021b; Wang F. et al., 2021; Chen et al., 2021b). In addition, numerous studies have demonstrated that dying cells, including ferroptotic cancer cells, communicate with the immune cells in the TME *via* a series of signals (Friedmann Angeli et al., 2019). These signals produced during cell death allow the recruitment and activation of immune cells, such as macrophages, regulatory T cell, and neutrophils (Matsushita et al., 2015; Klöditz and Fadeel, 2019; Li et al., 2019), which regulate the growth and expansion of other cancer cells.

**FIGURE 2:**
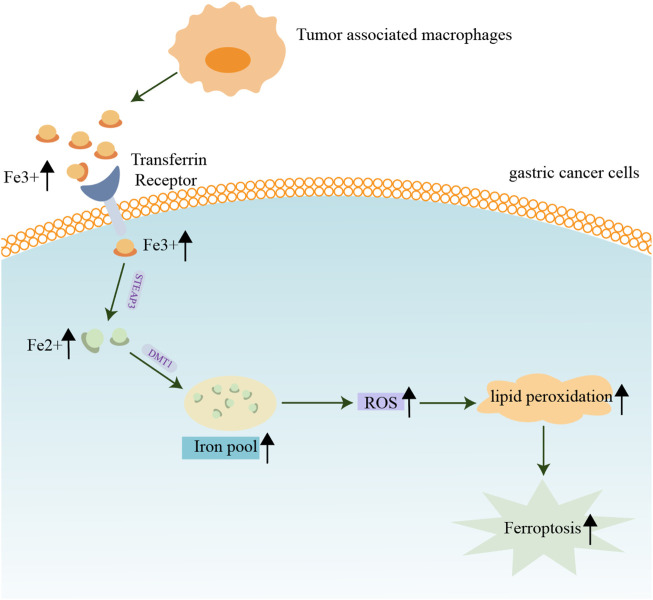
Contribution of iron and macrophages in the tumor microenvironment of gastric cancer. Macrophages maintain iron balance in human tissues. The proliferation of GC cells requires a large amount of iron, and the increased iron flow from tumor-associated macrophages promotes tumor progression or tumor protraction.

Tumor-associated macrophages are emerging as key players in the development of GC (Gambardella et al., 2020). Aside from its role in phagocytosis of foreign antigens, another physiological function of macrophages is to maintain the iron balance in human tissues. Iron homeostasis should be tightly maintained since excess labile iron is toxic (Henle and Linn, 1997; Muckenthaler et al., 2017). Surprisingly, malignant cells can evade the deleterious effect of excessive iron and require high amounts of these reactive ions for their proliferation (Pfeifhofer-Obermair et al., 2018). Depending on the circumstances, increased iron traffic by tumor-associated macrophages either promotes tumor progression or tumor protraction (Soares and Hamza, 2016). Therefore, the detection of macrophages and iron levels in the TME may provide a basis for predicting tumor progression (Liu S. J. et al., 2021; Xiang et al., 2021) (Figure 2). To better understand the functional role of ferroptosis and immune cells in TME, a comprehensive investigation of ferroptosis-related signals and the immune responses they trigger is warranted.

Despite of reduction in the incidence of GC and the development of novel therapeutic strategies, the prognosis of GC remains poor (Lazăr et al., 2018). Biomarkers for the characterization of the tumor immune microenvironment may add to the predictive value of the current staging system (Jiang et al., 2019). In recent decades, large-scale clinical trans-omics studies allowed the identification of some crucial ferroptosis-related genes as reliable biomarkers to describe the tumor immune microenvironment landscape and predict response to antitumor therapy (Liu S. J. et al., 2021; Shao et al., 2021).

### Drug Resistance in Gastric Cancer

Resistance to cisplatin and paclitaxel has become increasingly severe in GC patients (Shao et al., 2019; Zhai et al., 2019). This proves to be a major hurdle in clinical oncology and leads to poor prognosis (Chen Z. et al., 2020; Wei et al., 2020). Resistance to chemotherapy is usually related to mutations in genes regulating cell apoptosis and increased levels of glutathione (Silva et al., 2019). Interestingly, ferroptosis inducers may help in overcoming drug resistance and warrants further investigation (Shin et al., 2018).

**FIGURE 3:**
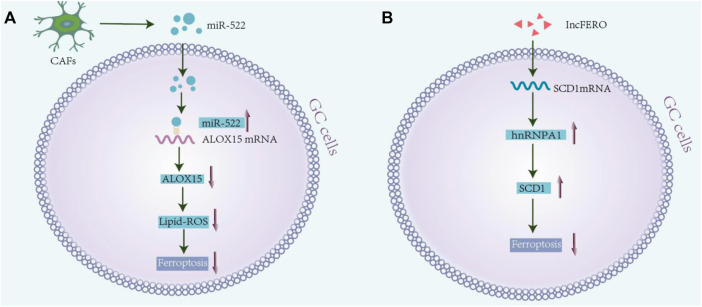
Drug resistance and ferroptosis in gastric cancer. **(A)** Exosomal mir-522 secreted from cancer-associated fibroblasts (CAFs) enter the GC cells and bind to ALOX15 mRNA, resulting in ALOX15 inhibition and reduction in lipid-ROS accumulation in cancer cells. It inhibits ferroptosis in GC cells, and finally reduces chemosensitivity (Namee and O'Driscoll, 2018). **(B)** Exosomal lnc-ENDOG-1:1 from GC cells can promote the expression of SCD1 by directly interacting with the SCD1 mRNA in GC cells and recruiting heterogenous ribonucleoprotein A1 (hnRNPA1), thereby leading to the inhibition of ferroptosis in GC cells.

Owing to genetic alterations and abnormal growth, cancer cells have higher oxidative tolerance from ROS than non-malignant cells. This ability is attributed to the maintenance of high levels of the antioxidant GSH, which is essential for cell survival and proliferation (Cramer et al., 2017). Studies show that blocking CAF-exosomes–mediated lipid-ROS inhibition leads to increased levels of ferroptosis in cancer cells, which in turn enhances cell sensitivity towards chemotherapy (Zhang et al., 2020). Another potential target for GC therapy is through the blockage of the ROS-activated GCN2-eIF2α-ATF4-xCT pathway, a signaling cascade leading to mitochondrial dysfunction-enhanced cisplatin resistance (Wang et al., 2016). In addition, regulating ROS levels may serve as another novel therapeutic strategy, since ROS can disturb the cellular oxidative environment and induce cell death (Dharmaraja, 2017). In line with this, studies have shown that the antioxidant enzyme, peroxiredoxin 2, significantly sensitizes the AGS and SNU-1 cells towards cisplatin treatment by regulating the level of ROS (Wang et al., 2020). As chronic and exorbitant ROS levels instigate drug resistance (Liao et al., 2019; Xu et al., 2020; Zhu et al., 2020), regulating ferroptosis may be a useful strategy for targeting the drug-resistant tumor cells (Yang et al., 2017; Huang et al., 2019; Choi et al., 2020; Zhang et al., 2021). A possible relevant mechanism is presented in Figure 3.

## Conclusion

Gastric adenocarcinoma is a common disease worldwide. Currently, surgery is the only considered effective treatment strategy. However, disease recurrence is very common even after complete resection (Johnston and Beckman, 2019). Interestingly, ferroptosis has been found to have a very vital role in several cancer types, especially in GC (Lee et al., 2020). As a relatively new discovered mode of cell death, the field of ferroptosis is a research hotspot. Although numerous studies have examined the biological mechanisms underlying ferroptosis, its relationship to tumor progression remains to be poorly understood.

In this review, we have highlighted the importance of iron metabolism and ferroptosis in GC. Iron is an important nutrient in humans (Tan et al., 1997; Goddard et al., 2011). However, iron oxidation also contributes to tumor formation and development of cancer (Doll et al., 2019). In addition, iron in macrophages of the tumor microenvironment is an important index for predicting and detecting GC as well as for evaluating the clinical utility of the related gene signature. Meanwhile, ferroptosis is an iron-dependent form of cell death, which is often characterized by the accumulation of lipid peroxidation products in a cellular-iron–dependent manner (Stockwell et al., 2017; Tang et al., 2021). It functions through two main pathways: iron metabolism and Xc system–induced ROS production (Zheng and Conrad, 2020). Different inducers can affect different steps in ferroptosis to regulate GC proliferation, invasion, and metastasis. Furthermore, the development of drug resistance in GC cells poses a major hurdle. As chronic and exorbitant ROS levels instigate drug resistance, ROS homeostasis may provide a useful treatment strategy for targeting the drug-resistant tumor cells.

Taken together, this review tries to elucidate the relationship between ferroptosis and GC, based on available research findings. We summarized the known ferroptosis processes mediated by gastric cancer-related biomolecules and discussed the actions of some drugs in the different pathways involved in ferroptosis. Lastly, this may serve as a reference for future studies on the mechanism of ferroptosis and the treatment of GC.

## Abbreviations

CAFs, cancer-associated fibroblasts; DMT1, divalent metal transporter 1; GC, gastric cancer; Gpx4, glutathione peroxidase 4; ncRNAs, noncoding RNAs; PUFA, polyunsaturated fatty acid; ROS, reactive oxygen species; STEAP3, six transmembrane protein of prostate 3.
